# SOS: speed of stomata opening and closing is influenced by vapor pressure deficit

**DOI:** 10.1093/plphys/kiae066

**Published:** 2024-02-06

**Authors:** Alexandra J Burgess, José Manuel Ugalde

**Affiliations:** Assistant Features Editor, Plant Physiology, American Society of Plant Biologists; Agriculture and Environmental Sciences, School of Biosciences, University of Nottingham, Sutton Bonington Campus, Loughborough, LE12 5RD, UK; Assistant Features Editor, Plant Physiology, American Society of Plant Biologists; INRES-Chemical Signalling, University of Bonn, Friedrich-Ebert-Allee 144, 53113 Bonn, Germany

Stomata, small pores on the surface of above-ground plant tissues, are critical for all aspects of plant physiology and metabolism (Lawson and Blatt 2014). The stomata structure consists of two specialized guard cells, surrounding a central pore. The appearance of these guard cells differs, from kidney-shaped within dicots to dumbbell-shaped in the monocot grasses. However, both perform the same function: to control the exchange of gases between the plant and the atmosphere. When stomata are open, carbon dioxide (CO_2_) is able to enter the intercellular space for use in photosynthesis, and, simultaneously, water and oxygen (O_2_) are lost to the environment. Therefore, stomata are critical for determining both photosynthesis and water-use efficiency (WUE), the balance between carbon gained and water lost. As such, stomata represent key targets for crop improvement and water-use efficiency under climate change (Condon 2020).

The aperture (i.e. size) of the stomatal pore is a function of guard cell morphology and cell turgor pressure. Internal and external signals control this pressure, with environmental cues including high light intensity, low vapor pressure deficit (VPD), and low internal CO_2_ concentration triggering stomatal opening (Raschke 1975). The speed of stomata opening (SOS) is one of the most important determinants of carbon uptake and WUE in fluctuating environments (Lawson and Blatt 2014; Long et al. 2022), as evidenced in recent studies that genetically manipulate opening speed (e.g. Horaruang et al. 2022). Therefore, understanding the mechanistic control of stomatal opening under changing conditions is of critical importance.

Within the angiosperms and members of the fern family Marsilaeceae, there is mechanical linkage between the stomata and the surrounding epidermal cells. In these plants, guard cells achieve larger stomatal apertures by displacing the neighboring epidermal cells. Therefore, the overall aperture of the stomatal pore is a result of opposing pressures between the two sets of cell types. Previous studies indicate that within these species, the epidermal turgor pressure has a greater influence on overall aperture size (Buckley et al. 2003; Franks and Farquhar 2007). As such, when water status decreases, there is a transient opening of stomata, resulting in a temporary increase in water loss, prior to closure (Buckley 2005). Modeling studies indicate that when epidermal turgor pressure is low, stomatal opening in the light should be faster in species with mechanical linkage (Mott et al. 1999). Evidence for this is complex due to the wide range of different guard cell morphologies, with dumbbell-shaped guard cells predicted to achieve higher opening speeds. Nevertheless, the link between stomatal opening speeds and the presence of mechanical linkage has not previously been explored.

In this issue of *Plant Physiology*, Pichaco et al. (2024) tested if the mechanical interaction between guard cells and epidermal cells modulates the light-dependent speed of stomata opening under variable VPD conditions. The authors compared the stomatal opening speed, measured as stomatal conductance over time, between plant species that have the mechanical linkage advantage between guard cells and epidermal cells (using the angiosperms *Erythrina sandwicensis*, *Senecio minimus*, and *Umbellularia californica* and exceptionally the fern, *Marsilea minuta*), against plants that do not have this mechanical linkage advantage (the gymnosperms *Callitris tuberculate*, *Xanthocyparis vietnamensis*, and the fern *Pteris vittate*; Fig. 1A). Their results show that a higher VPD increases the stomatal opening speed in the light only in plants that have a mechanical linkage between the guard cells and epidermal cells. On the contrary, plants that do not have this mechanical interaction did not show any difference in their stomata opening speed, regardless of local VPD (Fig. 1B). Moreover, after analyzing the variety in stomata anatomy among the species with mechanical interaction, the authors found a direct correlation between the ratio of guard and epidermal cell size and the light-dependent stomata opening induced by VPD. Plants with a smaller guard cell/epithelial cell ratio, such as *M. minuta*, have the greatest increase in stomata opening induced by VPD. In comparison, plants with a larger guard cell/epithelia cell ratio, such as *U. californica*, had slower stomata opening after an increase in VPD (Fig. 1C). The latter is an important observation since there are only a few studies that have linked physiological functions to the noticeable range of diversity in epidermal anatomy.

**Figure 1.**
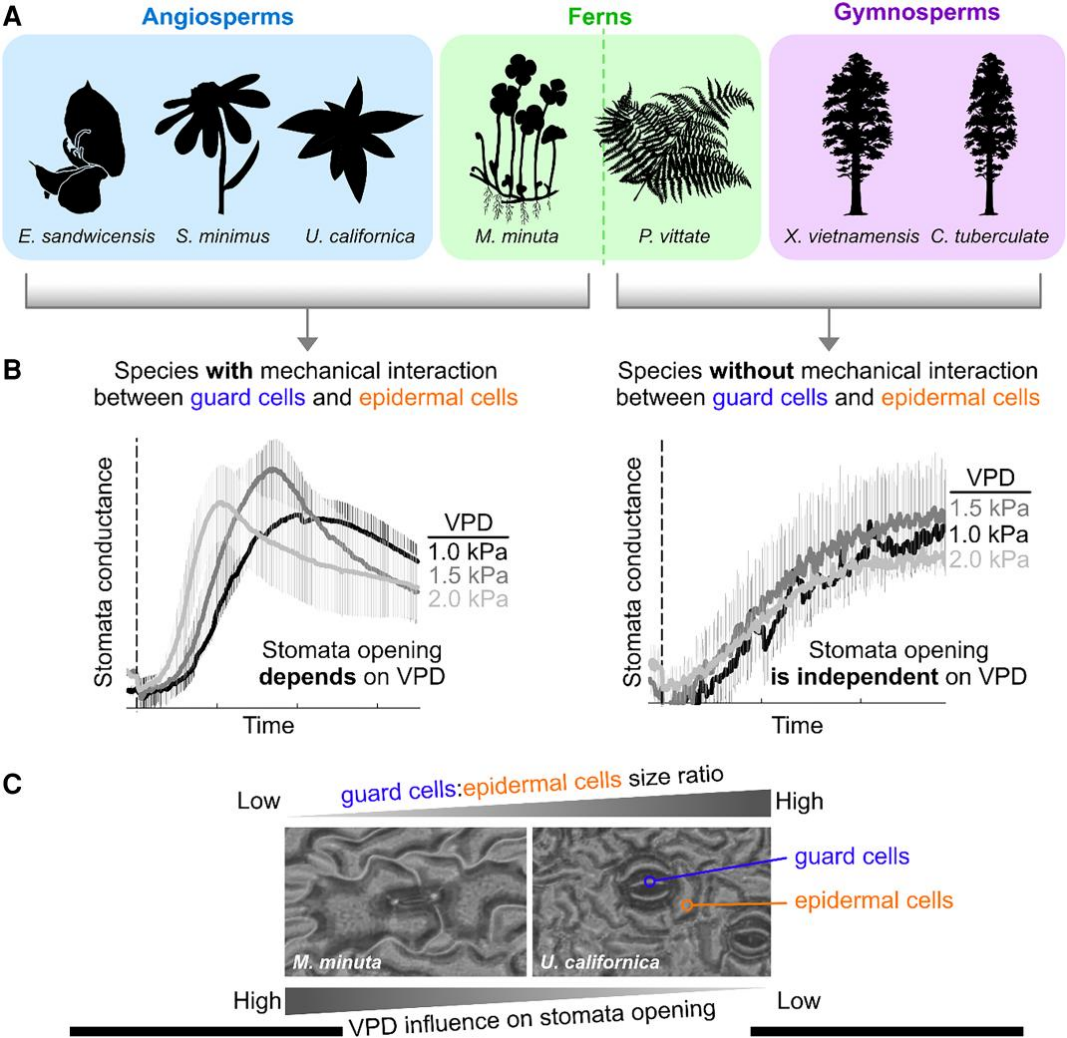
Stomata opening speed dependency on vapor pressure difference (VPD). **A)** Examples of plant species that have a mechanical interaction between guard cells and epidermal cells: angiosperms *Erythrina sandwicensis*, *Senecio minimus*, *Umbellularia californica*, the fern *Marsilea minut*a, and plants that do not have a mechanical linkage advantage: gymnosperms *Callitris tuberculat*e, *Xanthocyparis vietnamensis*, and the fern *Pteris vittate*. Most plant silhouettes were obtained from https://www.phylopic.org/. **B)** The dynamics of light-induced stomata opening was measured in the indicated plants as stomatal conductance over time under different VPD conditions. Plants with mechanical interaction displayed an increased speed of stomata (SOS) opening at higher VPD, while plants that did not have such interaction did not change their SOS opening upon exposure to different VPD conditions. **C)** Among the plants that respond to VPD changes, there is a direct correlation between the ratio of the guard cell size over the epidermal size. Plants with a lower ratio have a higher VPD influence (e.g. *M. minuta*), while plants with a lower ratio are less sensitive to changes in VPD (e.g. *U. californica*). Results in B and C are adapted from Pichaco et al. (2024). The figure was made by J.M.U using Affinity Designer version 2.3.1.

The results of Pichaco et al. (2024) support the hypothesis that decreased turgor pressure in epidermal cells increases stomata opening in a VPD-induced process. They established that the VPD dependency of light-induced stomata opening is limited to plants that have mechanical interaction between guard cells and epidermal cells, such as angiosperms and, exceptionally, the Marsilaeceae ferns (Fig. 1). Furthermore, the anatomy of the epidermis might be a determinant factor of the effect that epidermal turgor has on stomatal responses. Since a comparison of the stomata opening speed between genotypes is traditionally measured under constant VPD, the findings of this work highlight the possible underestimation of stomata response, when not measured under variable VPDs. These characteristics will be highly important to consider when searching for plants able to respond rapidly to an ever-changing environment.

## Data Availability

No new data were collected as part of this article.
